# A Review on Remediation of Iron Ore Mine Tailings via Organic Amendments Coupled with Phytoremediation

**DOI:** 10.3390/plants12091871

**Published:** 2023-05-03

**Authors:** Sajeevee S. Sarathchandra, Zed Rengel, Zakaria M. Solaiman

**Affiliations:** 1 UWA School of Agriculture and Environment, The University of Western Australia, Perth, WA 6009, Australia; 2 The UWA Institute of Agriculture, The University of Western Australia, Perth, WA 6009, Australia

**Keywords:** biochar, compost, grasses, phytoremediation, tailings, topsoil, straw

## Abstract

Mining operations degrade natural ecosystems by generating a large quantity of mine tailings. Mine tailings remain in dams/open ponds without further treatment after valuable metals such as iron ore have been extracted. Therefore, rehabilitation of tailings to mitigate the negative environmental impacts is of the utmost necessity. This review compares existing physical, chemical and amendment-assisted phytoremediation methods in the rehabilitation of mine tailings from the perspective of cost, reliability and durability. After review and discussion, it is concluded that amendment-assisted phytoremediation has received comparatively great attention; however, the selection of an appropriate phytoremediator is the critical step in the process. Moreover, the efficiency of phytoremediation is solely dependent on the amendment type and rate. Further, the application of advanced plant improvement technologies, such as genetically engineered plants produced for this purpose, would be an alternative solution. Further research is needed to determine the suitability of this method for the particular environment.

## 1. Introduction

Mining, mineral processing, and extractive metallurgy are essential industries for metal production but generate huge amounts of tailings [1]. Mine tailings are by-products of separating valuable minerals from uneconomic materials [2,3] and are generally deposited in open-air tailings impoundments without any treatments for a long time [4,5]. The global mining industry produces billions of tons of tailings each year, with an estimated 14 billion tons produced in 2010 [3], which equals the annual volume of mineral production. Iron ores are complex in nature, and tailings generation is varied from place to place; in Western Australia, it is estimated that tailings generation is 2 tons per ton of iron ore [6]. There is no accurate information on the volume of tailings waste produced during the iron extraction process, but the generation of fines content in the waste could be 10 million tons per year, as reported in India [6,7].

Therefore, huge quantities of tailings are generated during iron extraction from the ore and disposed of into the surrounding areas, creating vast barren lands [8]. These types of tailings ponds damage local land resources and produce severe environmental pollution [4] spatially in terms of the storage area and temporally in terms of the long time scales over which tailings must be managed and rehabilitated [2]. However, mine tailings management is a crucial issue in mining operations because of the irreversible impacts of tailings. Mine tailings have been reported to create significant risks to human health and the environment [9,10]. The adverse effects of metals on plants, including oxidative stress, fluorescence, stomatal resistance, chlorophyll and photosynthesis, reproductive processes, seed germination, seed morphology and seed physiology, have been reported [11]. Moreover, human health may be at risk via exposure to mine tailings through dermal contact, unexpected inhalation and the ingesting of dust from tailings. Consumption of food crops grown on mine tailings is harmful, as some of the heavy metal(loid)s (As, Ni, Cd, Cr, and Pb) are carcinogenic and can cause problems in the skin, kidney, lungs, skeleton, and gastrointestinal tract [11]. Iron ore tailings in Australia generally have (*w*/*w*), SiO_2_ 57%, AlO_3_ 10%, TiO_2_ 1%, and Fe_2_O_3_ 25% [6]. As reported by [2], the physicochemical properties of tailings present many challenges to achieving physically and chemically stable landscapes.

This review intends to provide an overview of the iron ore mine tailings knowledge and to frame the importance of green solutions coupled with the modern concept of “amendments-assisted” in the context of iron ore mine tailings rehabilitation, and future perspectives that could be addressed in terms of using advanced technology. The methodology used was an open-access literature search through Google scholar with the main keywords being “iron ore”, “mine tailings”, “phytoremediation”, “compost”, “topsoil”, “biochar” and “straw” over the ten-year periods.

## 2. Physicochemical and Biological Characterisation and Strategies to Improve Iron Ore Mine Tailings

### 2.1. Physicochemical Characterisation of Iron Ore Mine Tailings

**Figure 1:**
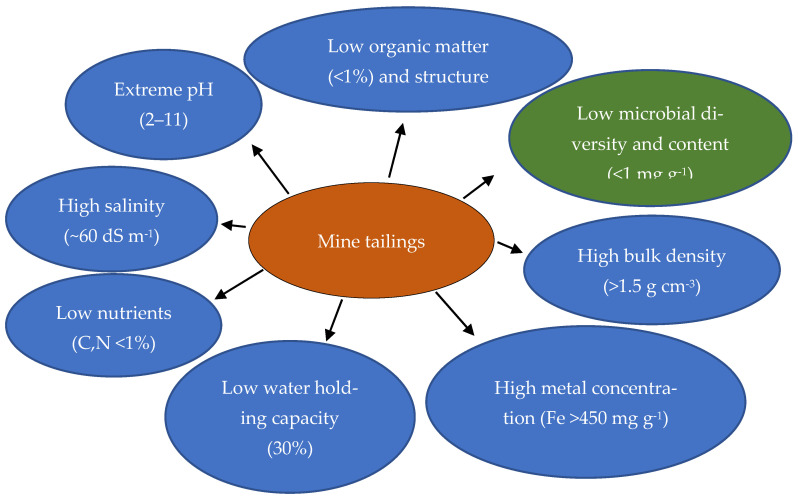
Schematic representation of physicochemical and biological characteristics of iron ore mine tailings.

Tailings often exhibit adverse physicochemical properties (Figure 1) such as poor structure [12] with a fine-grained blend of ground-up stone particles [13], low macronutrients, extreme pH [14], high salinity [15], high concentrations of metals, and residuals of processing chemicals [14]. Iron ore tailings are characterised by small particle sizes [4,5,12,16]. The content of heavy metals in tailings is influenced by many factors such as textural characteristics [17,18], mineralogical composition and depositional environment [19]. Numerous studies have emphasised the significance of soil properties such as particle size distribution and clay content on the behaviour of heavy metals in tailings [20,21]. Further to [21], metal mobility has been shown to be lower in fine- than in coarse-textured soils, especially if the mineralogical assemblage of the clayey soil is dominated by 2:1 tetrahedral: octahedral silicate clay minerals (e.g., illite or vermiculite with high cation-exchange capacities).

In some cases, iron ore tailings show high alkalinity, which reduces other nutrients [22] such as N, P, Ca and Mg availability [23]. The highlighted physical characteristics, such as poor water-holding capacity [17,24], low permeability [22], and high bulk density [17] make the situation critical. High bulk density of tailings increases the mechanical resistance, which limits aeration and reduces the infiltration of water and nutrients to the roots [25].

Low nutrient levels and potential ionic stress result from high solute concentrations [8,26] such as Si, Al, Ca and Mg [27]. Iron ore tailings are iron-bearing silicates consisting of silica skeletons formed by (SiO_4_) tetrahedron and forming a complex composition and stable structure with Fe^3+^, Ca^2+^ and Mg^2+^, which indicates that iron ore tailings have the potential to serve as a sustainable material by regulating the framework structure [27], but not for plant growth. In addition, the high content of organic matter also can enhance metal adsorption, thereby reducing mobility in the environment; however, mine tailings lack organic matter [28] and nutrients, which makes them an infertile substrate for plant growth [17,29]. However, if the mine tailings are acidic, the acidity will decrease the soil exchange capacities of metal cations and increase metal solubility in the soil environment, making heavy metals more mobile [21].

### 2.2. Biological Characterisation of Iron Ore Mine Tailings

The soil microbial community has a fundamental role in organic matter mineralisation, which allows the recycling of nutrients [30]. Heavy metals affect the number, diversity and microbial activity of soil microorganisms and also slow down growth and reproduction [30]. Some studies suggest that heavy metals negatively influence soil microbiological activities such as growth, morphology and metabolism through functional disturbance, protein denaturation or the destruction of the integrity of cell membranes [31]. In contrast, a few studies indicate no correlation between microbial aspects and heavy metal concentrations [32]. However, the lack of soil microorganisms and soil animals [17,33] in mine tailings leads to less microbial activity and enzyme activities in tailings [34]. The reasons could be identified as a low organic matter [35] and poor plant growth, resulting in low levels of aerobic heterotrophic bacteria, which use organic matter as an energy source [36]. Bacterial enumeration and enzymatic activities showed an apparent deterioration of microbial communities in the mine tailings, since no viable bacterial cells were detected, and dehydrogenase enzyme activity was extremely low [37]. Further, [38] pointed out that microbial activity in metal-polluted bare soils is disrupted by the toxic effects on microorganisms due to the absence of vegetation cover. It is worth noting that [39] showed that microbial diversity and structure in the rhizosphere and in bulk soils from mine tailings were mainly influenced by the presence of pioneer plants and the environmental conditions, especially nutrient elements and Fe contents, alongside plant community colonisation. Respiration can be considered a general measure of microbial activity that provides a reliable, repeatable and scientifically sound assessment of microbial activity [40]. Generally, metal concentration increases adversely, affecting soil microbial biomass, respiration rate and enzyme activity [41].

### 2.3. Strategies to Improve Physicochemical and Biological Characteristics of Iron Ore Tailings

Developing a sustainable strategy for pollution control in abandoned mines is of the utmost importance in reducing harmful effects on the adjacent environment and human health [38]. The most commonly used strategies for the remediation of mine tailings mainly focused on physical and chemical techniques, as they take less time. However, it is reported that these techniques are expensive, environmentally disruptive, and damaging to soil structure and biodiversity [38,42,43,44].

To restore the fertility of the tailings in order to make them suitable for vegetation growth, physical remediation uses physical methods, which include covering them with new and semi-new soil for melioration, electro-remediation technology, and heat treatment [45]. However, of the above-mentioned technologies, one of the most commonly used, the covering of the ground with new soil, has achieved desirable results and attained agricultural soil quality standards in China [45]. Although this physical method has achieved some success in tailings restoration, it is very costly and requires much labour. In addition, there is a high chance of collapsing the system due to precipitation, slope gradient, slope characteristics, and cover thickness in the tailings area and the new soil collection area due to ecological destruction and disturbances [45].

Chemical remediation uses chemical methods to improve pollution control and tailings, including leaching, organic fertiliser, chelating, and fixing agents [4,45]. Some studies [45] suggested that the application of alkaline materials (CaCO_3_) to the tailings may increase tailings’ pH value and result in reduced phytoavailable heavy metals concentration in tailings, concomitantly increasing metal sorption by the soil [46]. The addition of phosphate-based materials such as highly soluble diammonium phosphate (DAP) has proven to be highly effective for immobilising Cd, Pb and Zn in soil [47].

However, chemical methods such as leaching/acid extraction and soil washing are identified as effective and efficient compared to physical treatments and are applicable as an intensive treatment for small tailings facilities with a heavy pollution load [4,48]; however, this method impoverishes the physical properties of the soil and becomes a major limitation in chemical remediation [4,33]. Table 1 compares the costs associated with physical, chemical and biological remediation methods [4,49] as of July 2022.

Biological remediation strategies exploit microbial metabolism to stimulate favourable biotic and abiotic reactions to transform toxic compounds into innocuous substances [50]. However, the inner interrelationship between microbial resistance to heavy metal ions and their remediation ability remains unclear [51]. The proposed mechanism is that heavy metal ions can be captured by functional groups in/on microorganisms, and some heavy metal ions can be transformed from a toxic form to a less harmful form through a redox state change by microorganisms in order to reduce the toxicity of heavy metals efficiently [51]. These methods exhibit several environmental, technical and economic advantages compared to conventional methods, and implementation is promising [50] and cheap [4]. In addition, appropriate and cost-effective ecological rehabilitation at metal mines is an essential environmental measure and practice for building/developing mines in a greener concept [52].

## 3. Phytoremediation

Recent studies showed phytoremediation is a safe and efficient way to handle heavy metals in soil [19,53]. This cost-effective [12,54] plant-based approach to remediation takes advantage of the remarkable ability of plants to concentrate elements and compounds from the environment and to transform various molecules in their tissues [55]. Studies showed that the cost of phytoremediation of one square meter of Pb-contaminated soil (AUD 0.1) is 50–65% less compared to conventional restoration methods such as excavation and landfill (AUD 0.2) [12]. Further to [28], introducing plants in such a disturbed environment can reduce the migration of metal(loids) through the ecosystem and help restore soil fertility. The time required for the restoration of mining tailing sites depends on several factors, including metal concentrations in tailings, heavy metal mobility, target metal final concentrations, plant biomass, and rates of plant growth [56].

However, traditional phytoremediation techniques lack large-scale applications because of many limitations [57]. As explained by [57], the conventional approach of phytoremediation (phytoextraction, phytostabilisation, phytovolatilisation and phytomining) has changed and a new concept has arisen, which is to combine conventional–modern techniques such as organic amendments-assisted phytoremediation [57].

**Figure 2:**
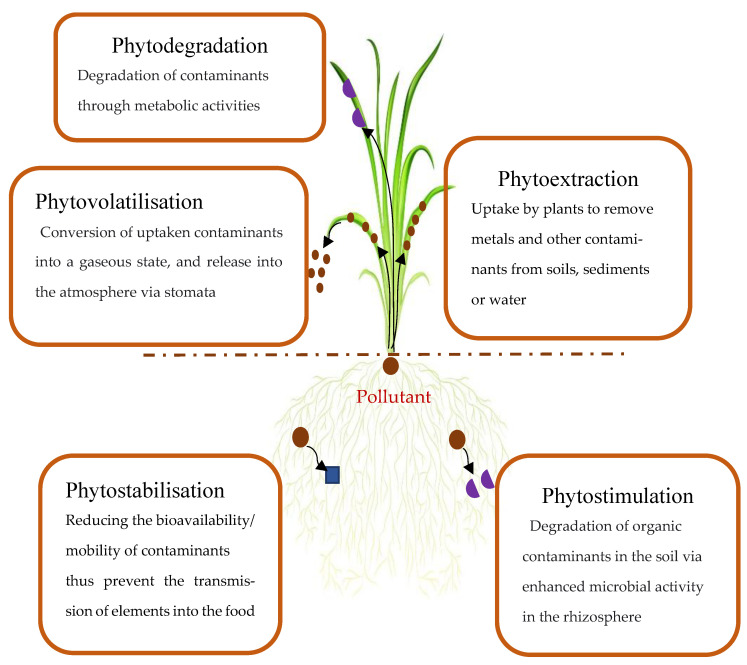
Schematic representation of different pathways of phytoremediation.

The plants and their associated microbiome can extract, degrade or immobilise pollutants from contaminated soil and water, and improve the ecological environment of the damaged soil [58,59], but not metals. Toxic heavy metals and organic pollutants are the primary targets for phytoremediation [55,57]. Chaney first suggested the concept of phytoremediation [57]. The phytoremediation techniques include phytoextraction, phytofiltration, phytostabilisation, phytovolatilisation and phytodegradation [60] (Figure 2). However, phytoremediation of mine tailings should include either inoculation of microorganisms and/or amelioration of organic amendments coupled with a phytoremediator (Figure 2).

### 3.1. Phytoextraction

Phytoextraction is the most important phytoremediation approach for removing metals(loid)s from contaminated soils, water, biosolids and sediments [61]. This technique uses uptake by plants to remove metals and other contaminants from soils, sediments or water, and it seems to be a simple and economical technique for the remediation of metal-polluted soils [62]. Successful phytoextraction requires that pollutant concentrations be reduced to a level that complies with environmental regulations. From an economic viewpoint, this should be achieved at a lower cost than an alternative technology or the cost of inaction [63]. However, it is stated that successful phytoextraction can be guaranteed by removing the time constraint of the operation, and any costs of phytoextraction incurred will be increased in proportion to the time taken [64].

Many factors affect the efficiency of phytoextraction, including soil properties, metal availability to plants, metal speciation and phytoremediator characteristics [57]. Therefore, the plant species/genotype selected for phytoextraction should possess the characteristics of (i) rapid growth rate that creates large biomass production, (ii) hyperaccumulator of heavy metals that translocates metal from root to shoot, (iii) tolerance to the toxic effects of heavy metals, [35] resistance to pathogens and pests, (v) good adaptation to prevailing climatic conditions, ease of cultivation and harvest, and (vi) no attractiveness to herbivores, to avoid the metal entry into the food chain [57,65].

Shoot metal concentration and shoot biomass mainly determine a suitable plant species for the phytoextraction of metals. Depending on these parameters, two different phytoextraction approaches have been used: (i) hyperaccumulators with relatively low shoot biomass, and (ii) use of plants with lower ability to accumulate heavy metals but with higher shoot biomass [66]. Further to [66,67], metal translocation to shoots is a crucial biochemical and physiological process, and it is desirable in effective phytoextraction because the harvest of root biomass is generally not feasible.

### 3.2. Phytostabilisation or Phytoimmobilisation

Phytostabilisation allows vegetation to be established on the surface of polluted soils/tailings by reducing the bioavailability/mobility of contaminants and their availability and thus preventing the transmission of elements into the food chain [68] by certain mechanisms including adsorption by roots, precipitation, and complexation in the root zone [57,66,69]. Phytostabilisation can be enhanced by using organic soil amendments that immobilise metal(loid)s when combined with plant species tolerant of a high level of contaminants while simultaneously improving the physical, chemical and biological properties of mine tailings [70]. Metals of different valences showed varied toxicity levels. Plants can convert those hazardous metals to a relatively less toxic state and decrease possible metal stress and damage by excreting redox enzymes [66]. In addition, phytostabilisation limits the accumulation of heavy metals in biota and minimises their leaching into underground waters [66]. However, this technique is not a permanent solution because the heavy metals remain in the soil; only their movement is limited [66]. However, it is a management strategy for stabilising (inactivating) potentially toxic contaminants [71].

## 4. Phytoremediation with Amendments

### 4.1. Phytoremediation with Biochar Amendment

Biochar is a fine-grained [72] carbonaceous porous substance synthesised as a result of pyrolysis of organic feedstock such as plant materials, organic manure and sludges [73] and was first discovered in the Amazon basin [74]. It has been postulated that biochar is an inert substance that could be a beneficial soil amendment for increasing soil quality and permanent mechanisms for carbon (C) sequestration to reduce CO_2_ enrichment of the atmosphere from anthropogenic activities [75,76]. Properties of biochar produced in slow pyrolysis tends to be similar to that produced by fast pyrolysis, which is a high-temperature process in which biomass is rapidly heated in the absence of oxygen. Furthermore, biochar can also be generated by gasification and hydrothermal carbonisation [77].

**Figure 3:**
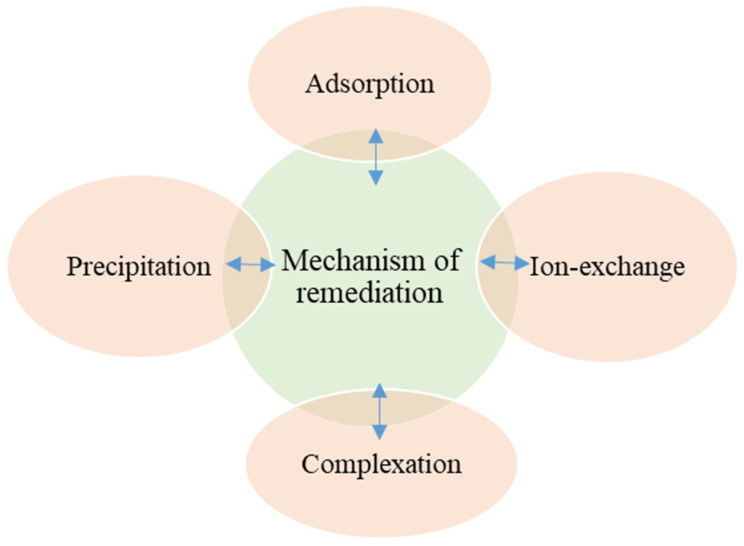
Heavy metal remediation mechanisms of biochar.

Chars are a more chemically heterogeneous group [75]. Still, the biochar produced by both methods has standard features regardless of feedstock source or synthesis. Biochar possesses some specific, unique physicochemical properties, i.e., extreme pH, large surface area for sorption of metals, carbon content and the ability to immobilise toxic heavy metals [57], and it shows the promising direction towards heavy metal remediation [78]. The high pH and alkalinity of biochar may decrease the bioavailability of metals and increase their precipitation in soil amended with biochar (Figure 3). It has been reported that the pH of biochar increases with pyrolysis temperature, possibly due to an increase in biochar ash content (fixed carbon and volatiles of metals) [57,79,80,81].

Biochar can also be combined with traditional phytoremediation techniques to enhance their effectiveness against heavy metals, as biochar is widely reported to improve plant growth and biomass production by up to 10% [82]. This increase in plant biomass is attributed to the high nutrient and water-holding capacity (4 to 130%), cation exchange capacity (CEC), and high pH (<7.0) of biochar, which affects nutrient cycling and improves the nutrient turnover of plants [83]. Furthermore, biochar influences the porosity (14 to 64%) and mechanical strength by changing the particle surface area, the pore size distribution, the particle size distribution and the density and aggregation (wet aggregation stability by 3 to 226%) [84,85]. Due to its highly porous structure, including various functional groups, it has been shown to be effective in the adsorption of heavy metals, especially in aquatic systems [72].

In addition, some chemical substances available in biochar, such as ethylene glycol and propylene glycol, hydroxy-propionic and butyric acids, benzoic acid and o-cresol, quinones (resorcinol and hydroquinone), and 2-phenoxyethanol, are reported to influence the soil microbial community, possibly by favouring beneficial microbes and suppressing pathogens [86]. The effect of biochar amendments on soil respiration and CO_2_ emission could vary [19,87]. Biochar application to mine tailings does not affect microbial respiration and CO_2_ emissions, which is consistent with previous studies that showed no significant effect on CO_2_ emissions from different soil types and land uses when applying biochar produced at high temperatures [88], confirming the possible effect of the high recalcitrance of this material and its high C sequestration potential [89]. The effects of its application may last for an extended period, given that biochar is highly recalcitrant [90]. In addition, biochar also resists microbial degradation, and its contribution to the microbial community is minimal [85,91]. Further to [92], there was no effect of birch biochar on soil respiration from a wheat field reported in Southern Finland, and soil CO_2_ evolution in Swiss loam soil was shown to be unchanged after being amended with pine wood-derived biochar, but increased under grass-derived biochar amendment [93].

CO_2_ production after biochar application could most likely be due to the labile C fraction originating from the condensation of bio-oil during the cooling period followed by pyrolysis of the feedstock; hence, it would not originate from the stable C of the biochar [75]. However, some studies, in contrast, suggested that it depends on the soil type that uses biochar and the feedstock used to produce biochar. Some studies have shown that different feedstocks have significantly affected the properties of biochar (yield, ash, element content, functional groups, aromaticity, porosity and specific area etc.) [94] (Table 2). The biochar produced from woody biomass has a significant level of thermal stabilising capacity of lignin, which showed higher specific surface area and porosity [86]. Further, crop residue and organic waste biochars have a higher pH than woody biomass biochar [95].

The application of biochar significantly increased soil respiration, which might be attributed to the reduced toxicity of metals, and increased nutrients by de-bonding complexes and plant growth, thereby enhancing microbial activity and decomposition of soil organic matter [72]. This is supported by the study of [75], which stated that they observed increased CO_2_ production from soils after biochar amendment from switchgrass *(Panicum virgatum*) feedstock, which increased when larger amounts of biochar were applied. Furthermore, [101] argued that biochar application to soil could repress the breakdown of native soluble organic carbon, often referred to as a negative priming effect. However, [19] concluded that small short-term C release in biochar-amended soil should not overshadow its potential for long-term C sequestration in soil environments, while [102] suggested that mechanisms for biochar-induced microbial activity simulations are yet to be discovered.

### 4.2. Phytoremediation with Compost Amendment

Compost production may be defined as the process whereby thermophilic, aerobic microorganisms convert organic materials such as plant residues, animal manure and bedding, or hay into pathogen-free, nutrient-enriched and biostable products [103]. To thrive and be most efficient in their work, the thermophilic microorganisms must be provided with temperatures of 50–70 °C. The inclusion of compost can facilitate the development of the arbuscular mycorrhiza (AM) symbiosis and improve plant growth even in extreme conditions in mine tailings [104,105]. Different studies showed the potentialities of compost derived from different feedstocks in improving the physicochemical characteristics of heavy-metal-contaminated soils and on intensively cultivated areas (Table 3).

Compared with inorganic materials, amendments such as composts have been proven to buffer soil pH, thereby indirectly affecting the adsorption and complexation of metals in mine tailings [4,106]. Additionally, compost could improve soil properties [105], nutritional status, water infiltration and water-holding capacity [33], ultimately having a positive effect on crop growth [107]. Phytoremediation of mine tailings by adding compost (15% and 20% *w*/*w*) in Arizona and the results demonstrated that canopy cover ranging from 21 to 61% developed after 41 months in the compost-amended planted treatments, while no plants grew on unamended tailings [108].

Compost from urban residues can be an adequate alternative to fertilisers because it provides organic matter and nutrients to the soil and stimulates the soil microbiological activity [109]. However, in barley (*Hordeum vulgare*), compost from sewage sludge application (4 kg m^−2^) induced a significant decrease in chlorophyll content in leaves due to a higher level of As translocation from roots to shoots [109].

**Table 3:** Summary of compost production with different feedstocks in literature.

Compost Feedstock	References
Dairy manure	[110]
Poultry manure and horse bedding	[111]
Pig manure	[112]
Rice straw	[113]
Sewage sludge, swine manure, sawdust, mushroom residue	[114]
Sewage sludge, barley straw, wood chips	[115]
Sewage sludge, kitchen waste and corn stalks	[116]
Municipal solid waste	[117]
Vegetable and fruit waste	[112]
Pine bark	[118]

### 4.3. Phytoremediation with Topsoil Amendment

According to [119], topsoiling may be necessary given the adverse physical and chemical properties of mine waste, the economics of properly amending these materials, and/or state and federal regulations. Some researchers advocated the placement of good quality topsoil over acidic mine waste, as the addition of topsoil may improve the water-holding capacity and nutrient status of the mine tailings and provide a source of reproductive parts and soil microorganisms [120]. However, one of the critical challenges faced in the post-disturbance restoration of arid lands, particularly post-mining, is the limited availability of topsoil [121]. Other problems encountered are the appropriate collection (stripping or stockpiling), storage and redistribution of topsoil, which are critical to maintaining these benefits in restored ecosystems. Once stripped, the viable topsoil (2–100 mm), which contains the bulk of the seed bank, should be returned and spread immediately, as storing of topsoil significantly reduces the seed bank viability [122]. Further, topsoil should be spread to a maximum depth of 10 cm to optimise efficacy and should be selected according to the topography of the restored area [123].

A possible approach to overcome this deficit is the reconstruction of soil profiles using alternative substrates, including mine tailings generated during the mining process and, where topsoil is available, a blend of topsoil and waste material [120,122,124,125]. Further, [121] revealed that seedling growth and survival rates were similar in topsoil and a topsoil/waste material blend, highlighting the importance of topsoil addition in reconstructing soils to ensure plant recruitment of key native species. For instance, topsoil amendments (50% w/w) to the Pb/Zn tailings facilitated the survival and growth of plants, arbuscular mycorrhizal fungi and earthworms [126].

### 4.4. Phytoremediation with Straw Amendment

Crop straw is an organic material that enriches soil nutrients and has a low environmental footprint [127]. However, burning crop straw at every summer harvest or in later autumn and winter [128,129] causes severe atmospheric environmental pollution and threatens human health. Therefore, extensive use of crop straw has become an economical and environmental approach to recycling waste straw [129].

At present, straw returning is an advanced technology for soil cultivation and usage which can significantly improve and modify the physicochemical and biological attributes of cultivated soil [130]. Most importantly, the application of crop straw can reduce the bulk density and increase the total porosity of the soil, which could provide a good space for root growth [131]. Studies have shown that adding crop straw increased soil pH and improved the metal adsorption by ligands in organic matter. Crop straw applied to the soil produces various organic anions by decomposing. The carboxylation process of organic anions and the OH^-^ ligand exchange reaction can increase soil pH value [132]. However, as [133] explained, the crop straw decomposed under the action of microorganisms, resulting in a large amount of humic acid and organic acid, which decreased the soil pH value.

Crop straw exerted a positive impact by increasing soil organic matter content, especially that of soluble organic carbon (SOC) [134], promoting crop growth and increasing the microbial populations in the topsoil, thus improving the microecology of soil and crop yield [135,136]. Moreover, the inhibition effect of crop straw mainly relies on the decomposition products produced by soil microbes. In addition, a higher number of functional groups present on the surface of crop straw may cause complex metal ions [131]. Further to [137], after adding rice straw and wheat straw, the accumulation of Cd in maize shoots decreased by 70 and 67%, respectively.

## 5. Plants in Poaceae Family Phytoremediating Heavy-Metal-Contaminated Mine Tailings

Plants used for effective phytoremediation should be fast-growing, deep-rooted, easily propagated and should show high biomass production and be able to tolerate and accumulate the targeted metal [54]. Selection of appropriate plant species would be essential to ensure a self-sustainable vegetation cover. Generally, those species which can accumulate high concentrations of heavy metal in aerial parts are supposed to be better and are called hyperaccumulator plants. According to [138], a plant that accumulates Cu, Co, Cr, Ni and Pb by > 1000 mg kg^−1^ or Mn and Zn by > 10 mg kg^−1^ is considered a hyperaccumulator.

The effectiveness and efficiency of phytoremediation technologies largely depend on the physiological characteristics of the selected plants and the kind of pollutants [139]. However, most hyperaccumulators have only a small biomass [139] and there is no suitable use for these hyperaccumulators, which absorb many heavy metals and quickly release them to the surrounding environment again as they are directly burned or converted into bioenergy. However, they could be promising candidates for hyperaccumulators because they usually have large biomasses and strong stress resistance [139] and few of them are shown in Table 4.

The grasses capable of growing in many places with different adverse climate conditions and having massive and deep root systems [140] include, for example, vetiver grass (Vetiveria zizanioides), tropical grass (Brachiaria brizantha), Smilo grass (Piptatherum miliaceum), Italian ryegrass (Lolium multiflorium), and perennial ryegrass (Lolium perenne) [140]. Atriplex spp. is known as a pioneer plant on mine tailings in semiarid Western Australia as a phytostabiliser [141].

**Table 4:** Application of grass species in remediating metal-contaminated lands.

Grass	Remediating Metal	Accumulation in Shoots (mg kg^−1^)	Reference
Vetiver grass (*Vetiveria zizanioides*)	Pb^2+^,	300	[142]
Signal grass (*Brachiaria decumbens*))	Pb^2+^	70	[143]
Italian ryegrass (*Lolium multiflorium*)	Pb^2+^, Cd^2+^	350, 800	[144]
Perennial ryegrass (*Lolium perenne*)	Cu^2+^, Zn^2+^	15, 180	[145]
Bermuda grass (*Cynodon dactylon* (L.)	Pb^2+^	400–1200	[141]
Esparto grass (*Lygeum spartum* (L.)	Zn^2+^,	>4100	[146]
Giant reed grass *(Arundo donax*)	As^2+^, Cd^2+^, Pb^2+^	23,25,26	[147]
Amur silver grass *(Miscanthus sacchariflorus)*	Zn^2+^, Cr^2+^	320,100	[139]
Rhodes grass (*Chloris gayana)*	Mn^2+^, Fe^2+^	164, 830	[148]
Buffel grass (*Cenchrus ciliaris*)	Ni^2+^, Cu^2+^, Zn^2+^	100, 13,10,	[149]
Common reed (*Phragmites australis*)	As^3+^		[150]

Vetiver grass (*Vetiveria zizanioides* (L.) Nash), has been well documented to have a solid resistance to undesirable environments, to be able to survive in high concentrations of heavy metals [139] and to remediate oil shale disposal piles. Naturally colonised Bermuda grass (*Cynodon dactylon* (L.) Pers.) yielded promising results when grown in Pb accumulated mine tailings in Spain [141]. As reported by [146], metal accumulation of Esparto grass (*Lygeum spartum* (L.) Albardine), known to colonise acidic mine tailings sites in Mediterranean regions, was examined in plants collected from the field and a greenhouse study. *Arundo donax* and *Miscanthus sacchariflorus* are two promising perennial energy grasses due to their fast growth rate [139], very high biomass (30–40 t ha^−1^) [151], strong stress resistance (e.g., cold, drought, salt) [139] and high conversion rate into ethanol [152], and are used to remediate Zn and Cr. A study conducted using Rhodes grass (*Chloris gayana Kunth* cv. ‘Pioneer’) found that it is well suited to metalliferous mined land revegetation and would be highly effective in the tropical and subtropical areas in Australia [153]. Further, these authors reported that buffel grass (*Cenchrus ciliaris*) is used effectively to remediate the lands disturbed by coal mining in Australia.

As reported by [154], the plant species perennial ryegrass (*Lolium perenne*) and perennial ryegrass/lucern (*Medicago sativa*) mixture caused the greatest change in the rhizosphere bacterial community in petroleum-contaminated soil, and these changes contributed to the degradation of the petroleum hydrocarbons in the contaminated soil. Common reed (*Phragmites australis*) and switchgrass (*Panicum virgatum*) have been demonstrated to be efficient in the detoxification of terbuthylazine and atrazine, respectively [155]. Compared with most hyperaccumulators, ryegrass is preferentially used for phytoremediation because it grows extensively, is easy to manage, incurs high biomass, and can accumulate many toxic substances in the tissues due to its high tolerance to heavy metals [156] and is more economical than other species [157]. However, the use of grasses in phytoremediation is not yet widespread due to the lack of studies on their metal accumulation potential under field conditions [158].

## 6. Conclusions and Future Perspectives

Rehabilitation of mine tailings is a critical environmental management practice as it conserves the surrounding environment, including the water, soil and air. Biological rehabilitation methods are more cost-effective and long-term than physical and chemical ones.

However, traditional phytoremediation approaches face certain limitations, such as: (i) they require a long time to remediate the contaminated soil, and the phytoextraction ability of hyperaccumulator plants is limited due to low aboveground biomass production; (ii) a tiny fraction of metals is bioavailable, and this bioavailable concentration varies with soil pH, organic matter, competitive cations, calcareousness, etc., and is applicable to sites with low or moderate contamination; (iii) lack of knowledge about agronomy, breeding potential, the insect pests and disease spectrum; and (vi) any mismanagement or carelessness may result in food chain contamination [57,66]. Therefore, to avoid these circumstances, traditional concepts were modified to ensure the large-scale application of phytoremediation.

Therefore, the best way to initiate biological rehabilitation is to combine it with the application of organic amendments in order to maintain a sustainable plant cover. The modified traditional concept/approaches are more economically applied on large scales, as naturally occurring hyperaccumulators are generally fast-growing and produce relatively more harvestable aboveground biomass when assisted with organic amendments such as biochar, straw, compost and topsoil.

Mining is a profitable industry that governs and dominates many countries’ economies. Therefore, they would prefer to invest in rehabilitation projects to gain profit/revenue. In such cases, phytoremediation is the best approach/scenario for mining land restoration, as it involves profitable and sustainable land use.

Further, co-cropping the tailings-deposited facilities using indigenous/endemic plants together with non-endemic plants would be beneficial in the long-term, over which the non-native plants improve the growth of the native plants by removing heavy metals and aiding in soil formation. However, in tropical climates, non-native plants act as invasives, threatening the establishment and biodiversity of the local environment. Nevertheless, it is worth noting that non-native invasive species are strong phytoremediators, since they can resist and uptake heavy metals by growing fast in unfavourable climatic conditions. Therefore, the selection of plants for mine tailings rehabilitation should be the first concern.

Practically, a single approach is neither possible nor practical for strategic rehabilitation of mine tailings; therefore, future research should focus on combining different methods, including genetically engineered plants and bioremediation coupled with organic amendments in sustainable restoration. Further, mechanisms and biochemical pathways underlying phytoremediators and organic amendments used in mine tailings are still uncovered, and this gap should be addressed in future research.

## Figures and Tables

**Table 1:** Cost comparison of different remediation methods for mine tailings calculated as AUD$ from published findings of [4,49].

Technique	Cost (AUD$/ton)		Factors to Be Considered
	[4]	[49]	
Physical remediation	140–720	-	Transport/excavation/monitoring
Chemical remediation	140–720	90–420	Recycling of pollutants
Phytoremediation	7–60	-	Long-term monitoring

**Table 2:** List of metal pollutant removal rates of certain biochars derived from different feedstocks under field conditions.

Feedstock	Pyrolysis Temperature	Type of Metal Pollutant	Removal Rate (%)	Reference
Rice straw	500 °C	Cd^2+^	98%	[96]
Rice straw	420 °C	Pb^2+^	95%	[97]
Corn straw	400–600 °C	Pb^2+^, Cd^2+^	60%, 80%	[98]
Peanut shells and shea nut shells	700 °C	Pb^2+^, Cd^2+^, Hg^2+^	100%	[99]
Rice husk	500 °C	Cd^2+^, Pb^2+^, Zn^2+^	25%, 18%, 17%	[100]

## Data Availability

The data supporting the results and conclusions in this manuscript are included within the paper.
